# Nutrition therapy and critical illness: practical guidance for the ICU, post-ICU, and long-term convalescence phases

**DOI:** 10.1186/s13054-019-2657-5

**Published:** 2019-11-21

**Authors:** Arthur Raymond Hubert van Zanten, Elisabeth De Waele, Paul Edmund Wischmeyer

**Affiliations:** 1 Department of Intensive Care Medicine, Gelderse Vallei Hospital, Willy Brandtlaan 10, 6716 RP Ede, The Netherlands; 2 Intensive Care Unit, UZ Brussel, Vrije Universiteit Brussel, Brussels, Belgium; 3 Department of Nutrition, UZ Brussel, Vrije Universiteit Brussel, Brussels, Belgium; 4 Department of Anesthesiology and Surgery, Duke University, Durham, NC USA

**Keywords:** Protein, Calories, Overfeeding, Underfeeding, Autophagy, Mitochondrial dysfunction, Refeeding syndrome, Micronutrients, Enteral feeding, Parenteral feeding, Oral nutrition supplements, Exercise

## Abstract

**Background:**

Although mortality due to critical illness has fallen over decades, the number of patients with long-term functional disabilities has increased, leading to impaired quality of life and significant healthcare costs. As an essential part of the multimodal interventions available to improve outcome of critical illness, optimal nutrition therapy should be provided during critical illness, after ICU discharge, and following hospital discharge.

**Methods:**

This narrative review summarizes the latest scientific insights and guidelines on ICU nutrition delivery. Practical guidance is given to provide optimal nutrition therapy during the three phases of the patient journey.

**Results:**

Based on recent literature and guidelines, gradual progression to caloric and protein targets during the initial phase of ICU stay is recommended. After this phase, full caloric dose can be provided, preferably based on indirect calorimetry. Phosphate should be monitored to detect refeeding hypophosphatemia, and when occurring, caloric restriction should be instituted. For proteins, at least 1.3 g of proteins/kg/day should be targeted after the initial phase. During the chronic ICU phase, and after ICU discharge, higher protein/caloric targets should be provided preferably combined with exercise. After ICU discharge, achieving protein targets is more difficult than reaching caloric goals, in particular after removal of the feeding tube. After hospital discharge, probably very high-dose protein and calorie feeding for prolonged duration is necessary to optimize the outcome. High-protein oral nutrition supplements are likely essential in this period. Several pharmacological options are available to combine with nutrition therapy to enhance the anabolic response and stimulate muscle protein synthesis.

**Conclusions:**

During and after ICU care, optimal nutrition therapy is essential to improve the long-term outcome to reduce the likelihood of the patient to becoming a “victim” of critical illness. Frequently, nutrition targets are not achieved in any phase of recovery. Personalized nutrition therapy, while respecting different targets during the phases of the patient journey after critical illness, should be prescribed and monitored.

## Introduction

Advances in ICU care allow for prolonged survival by providing life-sustaining support, making previously nonsurvivable ICU insults survivable. Innovations in ICU medicine have resulted in yearly reductions in hospital mortality [1]. However, many ICU “survivors” are not returning home to functional lives post-ICU, but instead to rehabilitation or nursing home settings where it is unclear whether they ever return to a meaningful quality of life (QoL) [2]. An increasing number of patients who survive ICU are suffering from severe, prolonged functional disabilities [2, 3]. Many ICU patients are likely to be discharged to post-acute care facilities and incur substantial costs (~ $3.5 million/functioning survivor in the USA) [4]. Disabilities are common, as 65% of ARDS survivors suffer significant functional limitations [2]. Thus, … “are we creating survivors … or victims?”

In 2012, the post-intensive care syndrome (PICS) definition was agreed upon by Needham et al. as the recommended term to describe new or worsening problems in physical, cognitive, or mental health status arising after a critical illness and persisting beyond acute care hospitalization [5]. Since then, both governmental agencies and ICU societies have recommended giving priority to research addressing post-ICU QoL [6]. To improve functional and QoL outcomes, one essential, low-cost therapeutic strategy that can be rapidly implemented is the optimal provision of nutrition throughout the ICU stay and recovery period.

Proper timing of nutrition therapy and optimal dosing has been suggested as critical illness and recovery metabolism changes throughout a patient’s course and energy expenditure and nitrogen losses appear to vary over time [7]. Nutritional therapy is essential, since associations between adequate feeding and outcome have been reported [8]. Almost no information is available on metabolic and nutritional demands of ICU survivors, and known nutritional practices reveal a poor nutritional performance during ICU stay and after discharge [9, 10].

This narrative review provides practical guidance on nutrition therapy for the ICU, post-ICU, and long-term convalescence phases, based on recent literature and guidelines. The key role of personalizing and timing the provision of macronutrients (calories and proteins) will be discussed.

## Nutrition therapy during ICU stay

The European Society for Clinical Nutrition and Metabolism (ESPEN) recently published evidence-based guidelines on medical nutrition therapy for critically ill patients [11]. Early enteral nutrition (EEN) is recommended, as it is superior over delayed enteral nutrition (EN) and early parenteral nutrition (PN). There are only few reasons to delay EN (Table 1).

**Table 1:** Reasons to start and delay early enteral nutrition

Recommendations	Rationale
Recommendation 1: Start early enteral nutrition in all critically ill patients within 48 h, preferably within 24 h when there is no reason to delay enteral nutrition (see the following recommendations).	Early enteral nutrition is associated with lower risk of infections and preserves the gut function, immunity, and absorptive capacity.
Recommendation 2: Delay early enteral nutrition in case of enteral obstruction.	Feeding proximal of an obstruction will lead to blow-out or perforation.
Recommendation 3: Delay early enteral nutrition in case of compromised splanchnic circulation such as uncontrolled shock, overt bowel ischemia, abdominal compartment syndrome, and during intra-abdominal hypertension when feeding increases abdominal pressures.	Absorption of nutrients demands energy and oxygen. In states of low flow or ischemia, forcing feeding into the ischemic gut may aggravate ischemia and lead to necrosis or perforation.
Recommendation 4: Delay early enteral nutrition in case of high-output fistula that cannot be bypassed.	Enteral feeding will be spilled into the peritoneal space or increase the fistula production.
Recommendation 5: Delay early enteral nutrition in case of active gastrointestinal bleeding.	Enteral feeding will limit the visualization of the upper gastrointestinal tract during endoscopy.
Recommendation 6: Delay early enteral nutrition in case of high gastrointestinal residual volume (> 500 mL per 6 h).	This threshold is associated with poor gastric emptying and may increase the risk of aspiration. Prokinetics and postpyloric feeding can circumvent this problem.

Adapted from references [10, 11]

When to start EEN in patients in shock is a matter of debate; however, EN can be commenced after the initial phase of hemodynamic stabilization, and it is not necessary to delay EN until vasopressors have been stopped [12, 13]. In the NUTRIREA-II trial among severe circulatory shock patients, an increased risk of splanchnic ischemia and gastrointestinal intolerance was observed induced by “forced” EEN [14]. However, in recent post hoc analysis from NUTRIREA-II, higher levels of citrulline were observed after 3 ICU days (reflecting enterocyte mass) in patients on EEN, suggesting EEN is beneficial for the gut mucosa even in severe circulatory shock patients [15].

### Progressive administration of calories

Based on pathophysiological insights from metabolism in the early phase of critical illness, this phase is characterized by inflammation, increased energy expenditure, insulin resistance, and a catabolic response leading to generation of energy from stores such as hepatic glycogen (glucose), fat (free fatty acids), and muscle (amino acids). Feeding ICU patients is essentially different compared with feeding the healthy [16]. The endogenous energy production in early critical illness cannot be abolished by nutrition therapy, and therefore, a progressive increase over days is recommended to prevent overfeeding [17]. This is further illustrated by the associations between the percentage of caloric target achieved during (early) ICU stay and energy expenditure (EE) measured by indirect calorimetry. The U-shaped relations found by Zusman and Weijs suggest that an energy intake of 70–80% of the measured EE is optimal, whereas lower and higher intakes both are associated with increased mortality [8, 18].

This U-shaped association was less clear when the results of the PERMIT trial on permissive underfeeding versus normocaloric feeding or energy-dense feeding versus normocaloric feeding in the TARGET trial are interpreted [19, 20]. In both large randomized controlled trials (RCTs), no differences in relevant clinical endpoints after low, normal, or high caloric intake during early ICU stay were observed. It is important to consider that in these trials the protein intake was the same in the study arms. The results of these RCTs seem to contradict the findings of the observational studies. However, in the RCTs, energy targets were estimated by equations and were not based on indirect calorimetry. As equations are inaccurate, overfeeding and underfeeding may have occurred in both study arms. In the PERMIT trial, differences in caloric intake were limited (estimated at 11 vs. 16 kcal/kg/day) and possibly too small to detect differences [21]. Another speculative explanation could be that caloric groups in the TARGET trial were fed on the up- and downsloping part of the U-shaped relation and therefore no differences in mortality could be observed.

Available data suggest that early overfeeding should be prevented and that hypocaloric or normocaloric feeding does not confer major differences in outcome when protein intake is similar. Aggressive early caloric intake leads to more episodes of hyperglycemia and need for high-dose insulin therapy, as was observed in the TARGET and EAT-ICU trials [20, 22]. As prolonged caloric deficits should be prevented, accepting a limited deficit (20–30% in the first ICU week) seems to be optimal. To estimate the caloric target after the initial phase, indirect calorimetry is strongly recommended [11].

### Refeeding syndrome and hypophosphatemia

Although refeeding syndrome (RFS) characterized by electrolyte shifts in response to reintroduction of nutrition after a period of starvation is ill-defined and many definitions are used, it can be best identified in ICU patients by refeeding hypophosphatemia (drop below 0.65 mmol/l within 72 h after the start of nutrition therapy) [23–25]. Several studies have shown that caloric restriction to 500 kcal/day or less than 50% of target for 2–3 days is essential to prevent attributable mortality from RFS [24, 25].

### Why are proteins important during critical illness?

Beneficial outcomes of critical illness are positively associated with the patients’ muscle mass on ICU admission, the predominant endogenous source of amino acids [26]. Moreover, the catabolic response leads to reductions in muscle mass up to 1 kg/day during the first 10 days of ICU stay in patients with MODS [27].

Mechanistic studies have shown beneficial effects on the loss of muscle mass and muscle protein synthesis induced by the administration of higher dosages of protein [28]. Many observational studies have shown that the provision of more protein as compared with lower intake of protein is associated with reductions in morbidity and mortality [8, 29–33]. However, the number of RCTs on enhanced protein administration is low and studies only show limited effects on functional and clinical outcomes or are negative [22, 34–38]. More evidence to prove improved outcomes is urgently warranted [39].

Diverging or negative results may be a result of study design, the interactions with calorie administration and overfeeding, or refeeding syndrome or due to dose, composition, and timing of the intervention [28]. Recently also, studies, such as the PROCASEPT study, have suggested that effects of proteins on outcome may be different in sepsis patients compared with other ICU patients [18, 40].

### Timing of proteins and progressive administration of proteins

Another explanation could be that very early high-protein intake in a post hoc analysis of the EPANIC trial, studying early versus late supplemental parenteral nutrition (SPN), was associated with negative effects on outcome [41]. This was confirmed in the retrospective PROTINVENT study showing increased mortality in patients treated with high-dose proteins during the first 3 days, although patients with an average intake below 0.8 g/kg/day showed the highest 6-month mortality after adjustment for relevant covariates [42, 43].

**Fig. 1:**
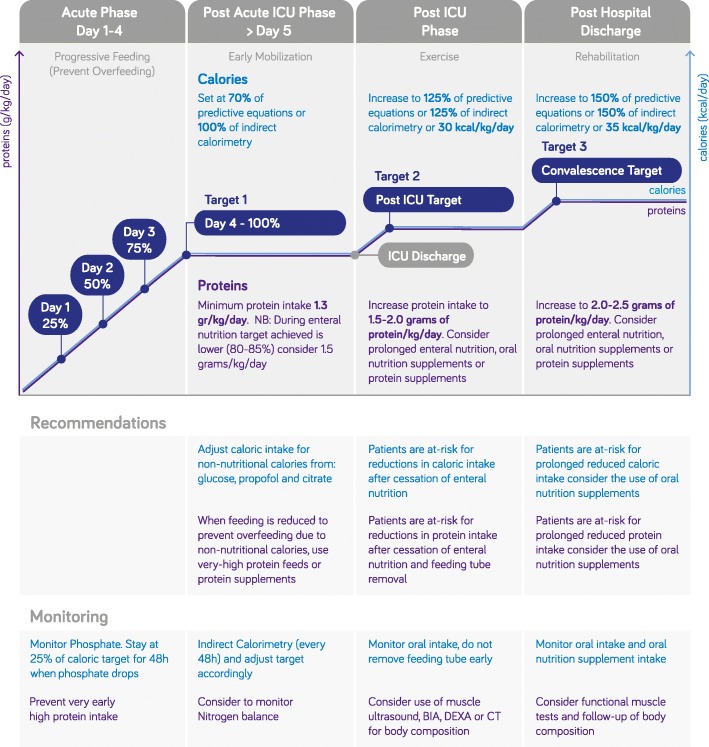
Practical approach to provide proteins and calories during the phases of critical illness and convalescence. g/kg/day grams of proteins per kilogram per day, kcal/day total kilocalories per day, BIA bioelectrical impedance analysis, DEXA dual-energy X-ray absorptiometry, CT computed tomography scanning. During the first 3 days, calories and proteins are gradually progressed to target 1 on day 4 in steps of 25% daily increase. Target 1 is 1.3 g/kg/day for proteins and for calories 70% of calculated targets or 100% of target when measured by indirect calorimetry. Target 2 should be met during chronic critical illness and after ICU discharge on general wards. For target 2, calories are increased to 125% of predictive equations or indirect calorimetry or 30 kcal/kg/day and for proteins 1.5–2.0 g/kg/day should be targeted. After hospital discharge, target 3 recommends a higher caloric target (150% of predictive equations or 35 kcal/kg/day) and a higher protein intake of 2.0–2.5 g/kg/day

Proteins and feeding in general are known to suppress autophagy, an important intracellular cleaning mechanism. Whether this should lead to the prevention of an autophagy-deficient state is a matter of debate [28]. Recently, a retrospective study did not show negative effects of early protein administration during ICU stay as it was shown to improve 60-day survival. In this study, moderate intake during the first 3 days was provided [44]. Based on the limited information and not to do harm, gradual progression to the protein target can be recommended [11, 45]. As this is also recommended for calories, step-wise increase to target in a few days can be performed using enteral nutrition (Fig. 1). Following the ESPEN guidelines, the protein target after progression should be at least 1.3 g/kg/day [11].

### How to reach the protein target?

A step-wise approach to meet the protein targets during critical illness is proposed to enhance a better performance (Table 2). This approach is based on the optimization of EN as a first step. However, it is challenging to meet the protein targets without overfeeding. Most tube feeds (and parenteral nutrition products) have a low-protein-to-energy ratio. Recently, a very-high-protein-to-energy ratio enteral feed based on intact protein was studied in an international randomized controlled trial compared with an isocaloric standard high-protein product [48]. With this new product, an average intake of 1.5 g/kg/day on day 5 was achieved, with a significantly higher amino acid concentration in the blood compared with the control product (mean protein intake 0.75 g/kg/day). The study clearly shows that using a standard high-protein product it is not possible to achieve intakes above 1.0–1.2 g/kg/day. Other ways to improve the protein intake is by using enteral protein supplements or supplemental amino acid solutions.

**Table 2:** Proposal to achieve a high-protein intake without overfeeding

Process step	Rationale	Reference
Step 1: Calculate the caloric need by your preferred equation and target 70% (first week) or measure energy expenditure by indirect calorimetry (after day 3) and set this as the 100% target.	Equations are inaccurate, and overfeeding is associated with increased morbidity and mortality. Early endogenous energy production cannot be inhibited by feeding.	[8, 11, 17]
Step 2: Subtract the amount of non-nutritional calories provided from propofol, glucose, or citrate.	Non-nutritional calories add to the total daily amount of calories and may lead to overfeeding when combined with full-dose feeding.	[46, 47]
Step 3: Calculate the daily limit for overfeeding (maximum calories allowed for feeding).	A step-wise build-up is recommended, for example, after ICU admission, go to target in steps of 25% to reach the target on day 4.	[11]
Step 4: Select a very high-protein-to-energy ratio enteral feed or the highest protein-energy ratio feed available and calculate the maximum acceptable dose based on step 3 without overfeeding.	Concentrated high-energy feeds increase the risk of overfeeding, while not meeting the protein target. When the protein ratio of total calories is higher than 30–32% in most patients, no additional protein supplements are needed.	[28, 48]
Step 5: Monitor the actual intake during the day and progress to higher than calculated infusion rates for limited time in case of previous interruptions of administration (stoppages), and use volume-based strategies.	There are many interruptions while feeding the critically ill; therefore, increasing the administration for short periods of time to compensate for the lost hours is a good strategy to meet the daily targets.	[49]
Step 6: Add enteral protein supplements in case more enteral feeding will lead to overfeeding when increasing the administration dose. Use no protein supplements during the very early phase (day 1–day 3).	In obese or overweight patients, the protein needs are very high while the caloric targets are not; then, even when using very-high-protein feeds, supplemental enteral protein supplements should be considered.	[11, 49]
Step 7: Add parenteral amino acid supplementation in case of contraindications to enteral feeding or inadequate enteral feeding/enteral protein supplementation at 4–7 day post-ICU admission (likely sooner in malnourished patients)	Whenever the enteral route is no option, consider the parenteral route.	[11, 49–51]

### Should we use intact proteins or hydrolyzed protein in the ICU?

Based on the available literature, there is no indication that pre-digested or hydrolyzed enteral feeds are better tolerated than intact protein feeds [52]. In some studies, the tolerance seems even worse and the target achieved lower compared with polymeric feeds [53, 54]. At present, recommendations are against the routine use of these semi-elemental formulations [49]. Whether semi-elemental formulations are superior in specific groups of patients at risk of enterocyte mass reduction and gut dysfunction, in particular patients with shock or sepsis, could be addressed in future studies.

### Timing of SPN

Early initiation of supplemental parenteral nutrition (SPN), before days 3–7, is not recommended based on a meta-analysis and recent guidelines [11, 50]. Only in patients with reasons to delay enteral nutrition and high nutritional risk early PN should be considered [11, 49]. SPN may increase infectious morbidity possibly due to the risk of overfeeding [55]. However, the recent TOP-UP trial suggested a benefit on Barthel Index-measured functional capacity (*p* < 0.08) in ICU patients at higher nutrition risk with low and high BMIs [51]. The role of earlier SPN use in malnourished or low BMI patients to improve functional outcomes requires further study.

### Monitoring of nutrition

No studies are available comparing outcomes with monitoring versus not monitoring nutrition therapy. However, the potential for abnormal values to be associated with harm was clearly recognized by a group of international experts [56]. Locally adapted standard operating procedures for the follow-up of EN and PN are recommended. Clinical observations, laboratory parameters (including blood glucose, electrolytes, triglycerides, liver tests), and monitoring of energy expenditure and body composition are essential to prevent and detect nutrition-related complications [56].

## Nutrition therapy during the post-ICU hospital stay

For this phase, no formal recommendations or guidelines on energy and protein intake are available. However, optimal caloric and protein intake is necessary to enhance recovery of functional muscle mass and to prevent further loss. It is very likely that significant calorie/protein delivery will be required to restore lost muscle mass and to improve QoL. Indirect calorimetry studies during the recovery phase demonstrate marked increase in metabolic needs, with total EE (TEE) increasing as much as ~ 1.7-fold above resting EE (REE) [57]. In the second week following sepsis, the TEE was 3250 kcal/day or 47 kcal/kg/day. In younger trauma patients, an even higher TEE 2 weeks post-injury of 4120 kcal/day or 59 kcal/kg/day was observed. In a retrospective study, a correlation was confirmed between higher protein delivery during ICU stay and survival: a decrease of 17% of 90-day post-discharge mortality rate was observed; however, no data on nutritional intake on the ward was accounted for [58].

Data on post-ICU protein targets are not available; however, considering that the average post-ICU patient is older and many of them are also frail, we may assume higher anabolic thresholds for protein synthesis (anabolic resistance). Therefore, an intake of 1.5–2.5 g/kg/day of proteins should be considered.

### How much is the nutritional intake post-ICU?

In post-ICU patients, a recent study reported an average spontaneous oral calorie intake of 700 kcal/day and the entire population studied consumed < 50% of calorie/protein needs for the post-ICU study period [59].

Another study evaluated 17 post-ICU patients during the hospital stay. The ward-based nutritional care showed to be of low efficacy and not in accordance with the existing recommendations. Several organizational issues were determined to be major barriers to optimal care [60]. A somewhat larger cohort study, including 32 patients, evaluated metabolic status and nutritional intake after ICU discharge [10]. The caloric daily need appeared to be around 2000 kcal and 112 g of protein. Intake was much lower, resulting in adequacy of nutritional therapy of 62% for calories and 54% for proteins. Patients were predominantly fed by the oral and enteral route. In those patients on oral nutrition alone without oral nutritional supplements (ONS), the intake was even lower (40%).

Recent unpublished data suggest that after removing the nasogastric feeding tube from post-ICU patients on the general ward the intake of calories drops by 22% and the protein intake by 27% of target (Van Zanten AR, personal communication). These data suggest that prolonged tube feeding until oral nutrition intake is sufficient should be considered as an alternative to usual care.

**Fig. 2:**
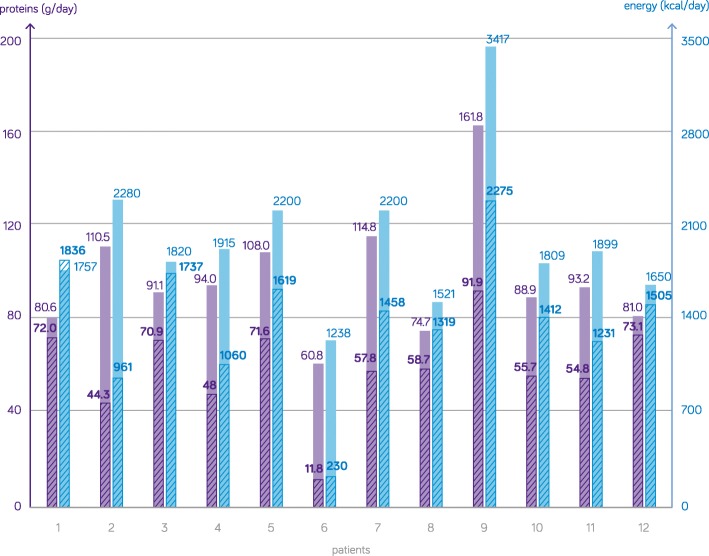
Average post-ICU nutrition intake (proteins and calories) related to individual targets. g/day grams of proteins per day, kcal/day total kilocalories per day. Full bars represent the mean calculated protein and energy targets, and the shaded areas represent the mean actual intake of protein and energy intake for each individual patient during the post-ICU observations days. Five female and 7 male patients with a mean age of 64 years and mean body weight of 75 kg were rather well fed during their ICU stay with a caloric adequacy of 86% of target for calories and 69% for proteins. As the initial days were also calculated during gradual progressing to target on the ICU, the objective can be considered lower than 100%. The calculated mean caloric need of the patients was 1967 ± 4519 kcal/day with only 66% of this target covered during the post-ICU phase on the general wards. Although 79 g of protein was mandatory, patients only received 62% of this daily amount during their ward stay. Large variability between patients is observed

Recent data from Brussels are in line with these findings: 12 patients discharged from ICU in 2018 were followed up during the entire hospitalization. Nutritional needs, prescriptions, and delivery were objectified. Adequacy of nutrition was calculated (Fig. 2, [61]). Large deviations were observed, predominantly underfeeding; however, also overfeeding was present. As ICU survivors spend more time outside than inside ICU, information on metabolic rates, nutritional adequacy, and effects of nutritional interventions are urgently needed. Caloric and protein intake of ICU survivors on the ward is low, representing clinically unacceptable low ratios of intake versus need.

### Nutrition rehabilitation after ICU discharge

After critical illness, restoration of the physiological regulation of food intake will improve over time. A wide array of functional alterations can hinder the intake of adequate amounts of nutrients during recovery. These alterations encompass changes in the preprandial phase, reflected by a loss of appetite; changes in the prandial phase, yielding swallowing disorders; and changes in the postprandial phase, including impairments of gastric emptying, gut motility, and satiety [62].

Further data on nutritional practices, barriers (e.g., high incidence of dysphagia after intubation), and possible solutions is urgently warranted. Although limited information is available, findings emphasize the importance of closely observing food intake in post-ICU patients before hospital discharge and instructing caregivers and healthcare professionals to provide optimal nutrition at home.

## Nutrition therapy after hospital discharge and convalescence

We must continue to consider whether patients leaving the hospital following an ICU stay will be able to consume adequate oral calories and protein to optimally recover at home or in rehabilitation facilities. Further, we must all take a moment to read and revel in the defining achievement that is the Minnesota Starvation Study and learn from its landmark lessons [7]. Even healthy subjects require significant calories (typically 3000–4500 kcal/day) and proteins up to 1.5–2.5 g/kg/day, to recover from the marked muscle loss that occurs following starvation.

In patients who have lost significant strength and muscle mass following an ICU stay, a considerable period of significantly increased calorie and protein delivery is required for recovery and likely needed for months to years [63]. Is it possible this lack of understanding has led to the extremely poor long-term outcomes and QoL.

How many of our care protocols, or our patients, will be able achieve this well-described goal without assistance from oral protein and nutrition supplementation? A large body of data demonstrates that oral nutrition supplement (ONS) must become fundamental to our post-hospital discharge care in ICU survivors. Meta-analyses in various hospitalized patients demonstrate ONS reduces mortality, reduces hospital complications, reduces hospital readmissions, shortens length of stay, and reduces hospital costs [64–67]. A large hospital database analysis of ONS use in 724,000 patients matched with controls not receiving ONS showed a 21% reduction in hospital LOS and for every $1 (US) spent on ONS, $52.63 was saved in hospital costs [68]. Finally, a recent large RCT of 652 patients studied the role of post-hospital high-protein ONS (HP-ONS) with β-hydroxy β-methylbutyrate (HP-HMB) versus placebo ONS in elderly malnourished (Subjective Global Assessment [SGA] class B or C) hospitalized adults. This definitive post-hospital trial demonstrates HP-ONS with HMB reduces 90-day mortality ~ 50% relative to placebo (4.8% vs. 9.7%; relative risk 0.49, 95% confidence interval [CI], 0.27–0.90; *p* = 0.018). The number-needed-to-treat in the post-hospital discharge setting to prevent 1 death was 20.3 (95% CI 10.9–121.4) [69]. As patients recovering from sepsis and the ICU will not consume sufficient calories and protein to recover optimally, the use of HP-ONS is essential and is strongly recommended for all ICU survivors post-hospital discharge for at least 3 months (and likely up to 2 years) following hospital discharge. In some patients, even prolonged tube feeding or parenteral nutrition should be considered.

### Key role for anabolic/anticatabolic agents

ICU survivors are also challenged by persistent catabolism and hypermetabolism for months to years. The HP-ONS trial and another recent review emphasize that anabolic and anticatabolic interventions, such as propranolol, oxandrolone, and other agents targeted at restoring lean muscle mass may be essential components to allow for meaningful recovery of QoL and survival post-ICU [69, 70]. Targeted nutrition that includes adequate protein delivery and “muscle-recovery targeted” anabolic/anticatabolic agents combined with exercise potentially lead to meaningful improvements in QoL [71].

### Propanolol

The data for the routine use of anabolic/anticatabolic agents in burn care is covered by a recent review [70]. Much can be learned from the vast experience with propranolol to reverse persistent hypercatabolism of critical illness [72]. This data showed that propranolol is the only intervention that will make a severely burned patient anabolic in the face of the largest and most severe catabolic insult humans can survive. Low-dose modern cardio-selective beta blockers to reverse catabolism are inadequate as was recently shown to have no impact on energy expenditure of ICU patients [73]. More research is warranted to evaluate the effect of propranolol in post-ICU patients.

### Testosterone and oxandrolone

Perhaps even more compelling is the growing body of literature for the safety, clinical efficacy, and benefit of testosterone and oxandrolone in a range of patients. It is well known that oral oxandrolone, among the most anabolic of the testosterone agents, is also among the safest as it shows minimal liver enzyme use with prolonged use. Oxandrolone has been shown to reduce mortality in burn-injured patients [74]. Concerns around potential cardiovascular risk and potential thrombotic risk have recently been dispelled in large observational studies such as the recent publication of > 43,000 subjects showing testosterone-deficient individuals (which virtually all ICU patients are within 7 days) on supplementation had a 33% reduction in all-cause cardiovascular events and a 28% reduced stroke risk [75]. A key recent meta-analysis showed that testosterone could improve exercise tolerance in heart failure patients [76]. It should be considered to check testosterone levels in patients in ICU 7 days or more, as they are often severely low or undetectable. Replacement may be done with testosterone cyprionate (~ dose 200 mg IM q 2 weeks), testosterone patch (~ dose 4 mg patch), or oxandrolone orally (~ dose 10 mg BID).

This is an area in desperate need of clinical trials outside of the burn setting as a meta-analysis of these pharmacological interventions to reduce ICU-acquired weakness did not find strong signals of benefit, except for the prevention of hyperglycemia during ICU stay [77].

## Conclusions

The interaction of acute metabolic changes, inflammation, and nutrition in early critical illness is complex. Newer insights suggest that progressive feeding in the early phase for both proteins and calories is essential to prevent overfeeding and high caloric intake during the development of refeeding hypophosphatemia. After this phase of 4–7 days, high-protein intake and sufficient calories are essential to prevent further loss of muscle mass and function.

After ICU discharge, the specific metabolic profile and nutritional needs of ICU survivors remain largely unknown and demand further research. Scarce data reveal poor nutritional practices for patients who left the ICU, are in the ward, and still have a long journey ahead of them.

Following hospital discharge, we must ensure our patients are complying with high-protein targets either by prolonged tube feeding or by enhanced high-protein oral nutrition (supplement) intake. Further, nutritional and metabolic therapies such as anabolic/anticatabolic agents in the recovery need urgent study.

But, to begin winning this war on long-term ICU outcomes and give our patients back the lives they came to us to restore, we must be thoughtful about optimal provision of nutrition and metabolic therapies throughout all phases of illness and ensure our patients are getting the right nutrition, in the right patient, at the right time!

## Data Availability

Not applicable
